# Are we facing an increasing surgical demand for high myopic traction maculopathies? A 12-year study from Hong Kong

**DOI:** 10.1186/s12886-022-02709-z

**Published:** 2023-01-23

**Authors:** Kenneth K. W. Li, Daniel H. T. Wong, Patrick S. H. Li

**Affiliations:** 1 Department of Ophthalmology, United Christian Hospital, Hospital Authority, 130 Hip Wo Street, Kwun Tong, Kowloon, Hong Kong SAR, China; 2 Department of Ophthalmology, Tseung Kwan O Hospital, Hospital Authority, No. 2 Po Ning Lane, Hang Hau, Tseung Kwan O, Hong Kong SAR, China; 3 Department of Ophthalmology, School of Clinical Medicine, LKS Faculty of Medicine, The University of Hong Kong, Hong Kong SAR, China

**Keywords:** High Myopia, Pathological myopia, Myopic traction maculopathy

## Abstract

**Purpose:**

We aimed to investigate the longitudinal change in the number of surgically operated myopic traction maculopathies (MTM) cases at a tertiary eye centre.

**Methods:**

A retrospective study of all consecutive cases of surgically operated MTM over 12 years (2009-2020) was conducted in a myopia prevalent region. We compared outcomes among three groups: (1) myopic macular hole (MH), (2) myopic macular hole with retinal detachment (MHRD), and (3) myopic foveoschisis with retinal detachment (MFRD).

**Results:**

Fifty-one cases were included in the study (8 cases of MH, 33 cases of MHRD and 10 cases of MFRD). The overall mean age was 63.8 +/- 8.7 with a female preponderance (2:1). The mean age of the MH group (58.6) was significantly younger than the MHRD group (64.2) and MFRD group (66.6) (*p* = 0.02). Subgroup analysis using ATN classification did not show its correlation with both visual improvement and anatomical success. When comparing the first 6-year period (2009-2014) with the second 6-year period (2015-2020), there was a significant increase in the number of cases (*p* = 0.01).

**Conclusion:**

We observe an increase in the number of surgically operated MTM. This follows the trend of the global rise in the prevalence of myopia and baby boomers entering retirement.

## Introduction

High myopia is known to lead to visual loss secondary to different types of maculopathy. Among these, myopic tractional maculopathies (MTM) are treatable with surgeries. Different pathophysiology of MTM have been described, including anteroposterior and tangential macular traction of the vitreoretinal interface, reduction of chorioretinal adhesion, and posterior staphyloma may contribute to tractional changes to the macula and even retinal detachment [1]. In 1999, retinoschisis was first described by Takano et al. using the time-domain optical coherence tomography (OCT) [2]. The syndrome myopic traction maculopathy was first described by Panozzo et al. in 2004 with the aid of OCT findings [3]. With recent advances in OCT utilising spectral-domain and swept-source technology (SD-OCT/SS-OCT), the reliability of its diagnosis has since improved. The current understanding is that MTM is a spectrum of diseases, namely myopic macular hole (MMH), myopic foveoschsis (MF) or macula hole-related retinal detachment (MHRD) [1, 4]. Various surgical techniques have been described, including internal limiting membrane peeling, macular buckling and scleral imbrication [1, 5–7].

Globally, the prevalence of myopia has risen dramatically over the past 50 years, and it is known that MTM increases with age, higher refractive error and longer axial length [8–10]. With increased life expectancy and baby boomers entering retirement age, it is plausible that vitreoretinal surgeons will face an increase in MTM cases. However, most studies in the literature focused mainly on the surgical success rate of the individual disease entity [5, 11]. There are limited epidemiological studies evaluating the prevalence of the three clinical entities of MTM in a longitudinal manner.

In the past, grading of MTM was not widely practised but relied mainly on clinical examination using slit lamp biomicroscopy or fundus photographs. Some classification systems based on fundus photography exist, for example, the International Photographic Classification Grading System [12]. With OCT machines becoming ubiquitous in clinical practice, diagnosis of MTM is now more accurate. Parolini et al. introduced the Myopic traction maculopathy Staging System (MSS) and classified two evolutional patterns based on OCT images [13]. They defined the mean evolution time of MTM, confirmed the visual acuity decreased with increasing stages and concluded that MTM staging and age were correlated. In 2019, Ruiz-Medrano et al. introduced the ATN classification based on clinical examination and OCT findings [14, 15]. They classified MTM by macular appearance (Atrophic, A), the presence of traction on OCT (Tractional, T), and the presence or history of neovascularisation (Neovascularisation, N). This system accounted for tractional and neovascularization-related alterations and was found to have high intraobserver and interobserver correlation [16].

With an increase in the prevalence of myopia globally, we want to study if there is an increase in surgical demand for this spectrum of disease. Therefore, we conducted a retrospective study of consecutive cases of surgically operated high myopia-related maculopathies over 12 years in a myopia prevalent region. We compare the surgical outcomes among the three groups of MTM and determine whether there was an increase in surgical demand over the study period. We also incorporated the ATN classification to see if predicting the prognosis of surgically operated cases is helpful.

## Methods

A retrospective study was conducted by including all consecutive cases of surgically operated high myopia-related maculopathies at United Christian Hospital, Kowloon, Hong Kong, over 12 years. Our centre provides tertiary eye care to the eastern peninsula of Kowloon in Hong Kong Special Administrative Region, China, with a catchment population of 1.1 million. The cases were retrieved using a combination of electronic health records and a surgical logbook. During the study, all patients received pars plana vitrectomy by two vitreoretinal surgeons (KL and PL). Only cases of myopic macular hole (MH), myopic macular hole with retinal detachment (MHRD) and myopic foveoschisis with retinal detachment (MFRD) were included. The definition of high myopia in the literature has been the axial length longer than or equal to 26.5 mm with or without a spherical equivalent of more than -6 Dioptres [17]. We adopted axial length longer than or equal to 26.5mm as the only criteria for high myopia. It was measured by biometry using either ultrasound or optical method (IOLMaster® 500 ZEISS, or Alcon OcuScan RxP Ophthalmic Ultrasound System). Other inclusion criteria included OCT evidence of macular hole with or without retinal detachment or foveoschisis with foveal detachment. Exclusion criteria included previous intraocular surgeries other than cataract extraction and cases with a follow-up period of fewer than six months. All patients underwent colour fundus photography (Nidek AFC-210, Nidek Co. Ltd, Gamagori, Aichi, Japan), infrared photography and spectral domain optical coherence tomography (Heidelberg Spectralis, Heidelberg Engineering GmbH, Heidelberg, Germany).

The anatomical success, defined as OCT evidence of macular hole closure, resolution of retinal detachment and foveoschisis, is determined by clinical examination and postoperative OCT at six months. Other outcomes include a change of LogMAR visual acuity at the one-year follow-up and preoperative staging of the maculopathies using ATN classification.

**Fig. 1:**
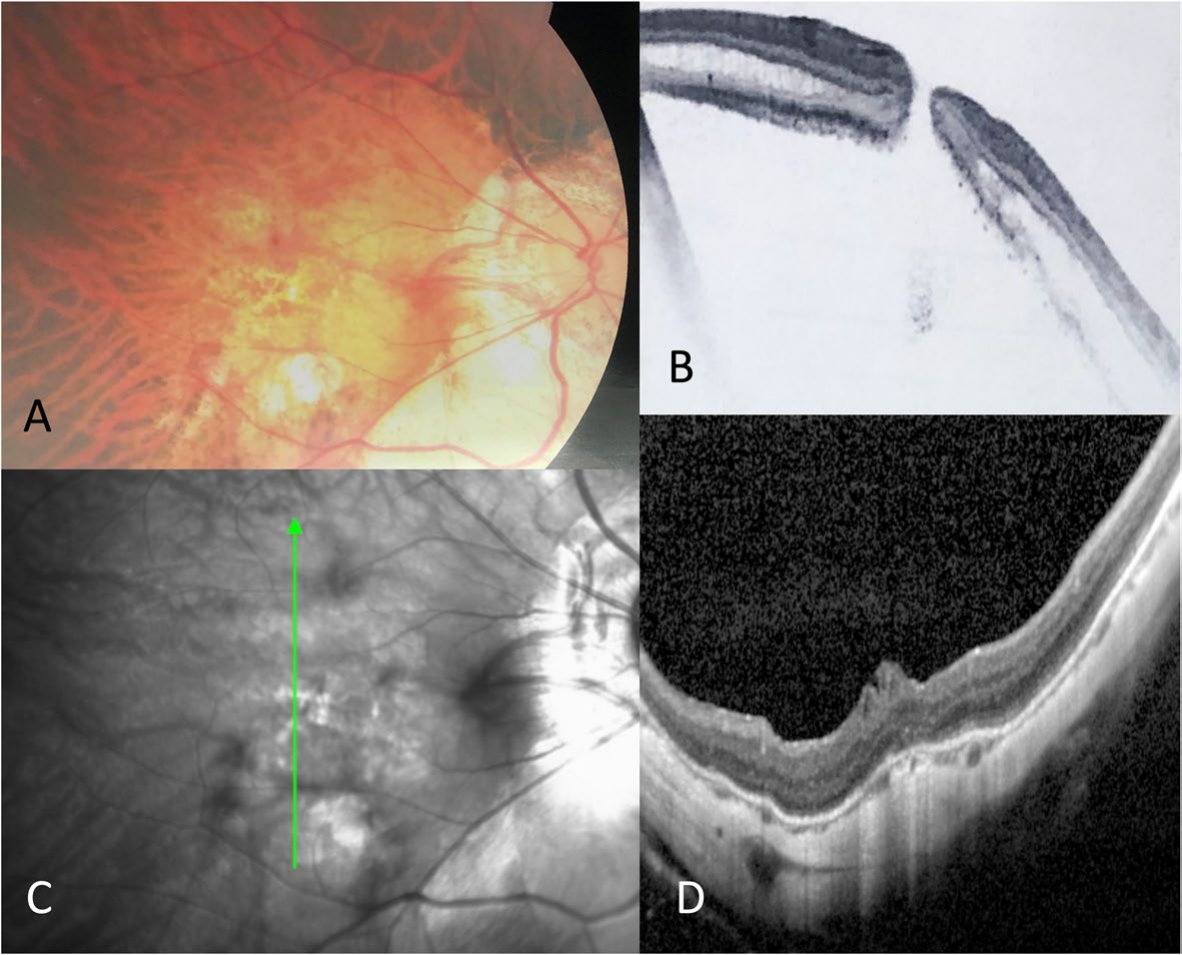
A case study of an operated MTM. A 61-year-old patient with right eye axial length of 32.5 mm, myopic macula hole with retinal detachment (A3T5N0) and a preoperative visual acuity of 0.05. The case underwent phacoemulsification, implantation of intraocular lens in the posterior chamber, pars plana vitrectomy, internal limited membrane peeling with inverted flap, and 12% C_3_F_8_ tamponade. The post op visual acuity improved to 0.2 and remained stable in the following 4 years. **A** Postoperative coloured fundus photograph. **B** Pre-operative optical coherence tomography (OCT) showing full-thickness macular hole with retinal detachment. **C** Postoperative infrared fundus image. **D** Corresponding postoperative OCT showed complete closure of macula hole with foveal thickening

For staging using ATN classification, a multi-source grading method (electronic patient records, fundus photographs, cross-section optical coherence tomography image and confocal scanning laser ophthalmoscope fundus image) was used. A case with the corresponding fundus photo and OCT was shown in Fig. 1. Not all cases had preoperative fundus photographs for grading atrophic “A” changes, so we assigned additional criteria to facilitate retrospective grading based on the clinical notes entry (Table 1). This included adding “characteristic not mentioned” to A0, “atrophic” or “myopic maculopathy” to A3, and “macular scar” or “extensive choroidal atrophy” to A4. Two independent observers reviewed the cases and images separately, and in case(s) of dispute, arbitration was conducted in the presence of a third independent observer. All of these were retinal specialists. Our three groups of myopic macular hole (MH), myopic macular hole with retinal detachment (MHRD), and myopic foveoschisis with retinal detachment (MFRD) corresponded to T4, T5 and T3 on the ATN classification, respectively.

**Table 1:** Modified ATN classification adopted in our study for reviewing clinical notes and images

Atrophic MM	Tractional MM	Neovascular MM
A0 – no myopic retinal lesions/ characteristic not mentioned A1 – tessellated fundus A2 – diffuse chorioretinal atrophy A3 – patchy chorioretinal atrophy/ “atrophic”/ “myopic maculopathy” A4 – complete macular atrophy / macula scar / extensive chorioretinal atrophy	T0 – no macular schsis T1 – inner or outer foveoschsis T2 – inner and outer foveoschsis T3 – foveal detachment T4 – full thickness MH T5 – MH and retinal detachment	N0 – no myopic CNV N1- macular lacquer cracks N2a – Active CNV N2s – scar or Fuch’s spot

*MM* Myopic maculopathy, *A* Atrophic, *T* Tractional, *N* Neovascular, *MH* macular hole, *CNV* choroidal neovascularisation

All cases of MTM were referred to the vitreoretinal clinic with their OCT reviewed and surgery offered by the two surgeons. The indication to offer surgery did not change during the study period. Surgery would be provided for patients who complained of decreased vision with a visual acuity of less than 6/12 presence of tractional myopic maculopathy, equivalent to T3 to T5 in the new ATN classification. The indication of surgery did not change over the period based on improvements in available diagnostics and surgical systems.

We also compared visual and anatomical outcomes among the three groups for any difference and determined if there has been an increasing demand for surgery over the past decade. Statistical analysis was performed using SPSS software; a p-value of less than 0.05 is considered significant.

## Results

51 cases met the inclusion criteria during the 12-year study period (January 2009 to December 2020) and were included. Patient demographics, changes in visual acuity, and anatomical success were summarised in Table 2. All cases had a successful pre-op and post-op OCT image; hence, all had the diagnosis confirmed with OCT. All cases had their medical notes, and photos where available, reviewed. Out of the 51 cases, there were 8 cases of MH, 33 cases of MHRD and 10 cases of MFRD. 4 patients had both eyes (*n* = 8) operated on. The overall mean age was 63.8 +/-8.6 (range 41 to 87) with female preponderance (2:1). The mean age was significantly younger in the MH group at 58.6 when compared with the MHRD group (64.2) and MFRD group (66.6) (Kruskal Wallis test, *p*=0.02). There was no statistical difference in the mean axial length among the three groups (MH 28.4, MHRD 29.6 and MFRD 29.8mm, *p*=0.08). The MH and MFRD groups had C3F8 as the exclusive or near-exclusive tamponade agent, whereas nearly half of the MHRD group had silicone oil (*p*=0.01). For postoperative LogMAR visual changes at one year, the MHRD group and MFRD group had visual improvement of +0.31 and +0.11, respectively, but the MH group had lost 0.11. The difference, however, was not statistically significant (*p*=0.12). For anatomical success, the MHRD group had the highest success rate at 78.8%, followed by the MH group at 75% and then the MFRD group at 70%. The difference again was not statistically significant (*p*= 0.89).

**Table 2:** Patient demographics, visual outcome and anatomical success

	myopic macular hole with no detachment (MH group)	myopic macular hole with retinal detachment (MHRD group)	myopic foveoschisis with retinal detachment (MFRD group)	*p*-value (if applicable)
Number of cases	8	33	10	-
Number of cases with gradable fundus image	5	24	8	-
Average age	58.6 +/- 3.4	64.2 +/- 7.9	66.6 +/- 12.1	0.023 ^a^#
Gender (M:F)	4:3	2:5	3:7	-
Mean axial length (mm)	28.4	29.6	29.8	0.118^a^
Change in LogMAR VA	-0.1	+0.3	+0.1	0.083 ^a^
Tamponade agent (percentage)	C_3_F_8_: 8 (100) Silicone oil: 0 (0)	C_3_F_8_: 17 (51.5) Silicone oil: 16 (48.4)	C_3_F_8_: 9 (90) Silicone oil: 1 (10)	0.007 ^b^#
Anatomical Success (percentage)	6 (75)	26 (78.8)	7 (70)	0.890^a^

^a^Kruskal Wallis test ^b^Chi-square test # *p* < 0.05

When considering atrophic changes in ATN classification, 37.5% of cases in the MH group, 24.24% in the MHRD group and 60% in the MFRD group were classified as A2 (diffuse chorioretinal atrophy). On the other hand, 12.5% of MH, 18.1% of MHRD and 10% of MFRD were classified as A3 (patchy chorioretinal atrophy/atrophic/myopic maculopathy). Whereas 12.5% in the MH group, 39.39% in MHRD and 60% in MFRD were classified as A4 (complete macula atrophy/macular scar/extensive chorioretinal atrophy), The majority (>80%) of all three groups did not have any history of choroidal neovascularisation. There was no statistical difference in the atrophic and neovascular components among the three groups (Table 3). We further stratified cases by high (grade 3 or above) and low (grade 2 or below) atrophic and tractional components on the ATN classification. Then we performed an analysis, comparing visual acuity gain and anatomical success with these two stratifications but failed to find any significant difference across the two groups (Table 4).

**Table 3:** Individual ATN classification of MH, MHRD and MFRD groups

ATN Grading		MH Group	MHRD Group	MFRD Group	*p* value
Atrophic (A)	0	3 (37.5)	6 (18.18)	-	0.2198
	1	-	-	-	
	2	3 (37.5)	8 (24.24)	6 (60)	
	3	1 (12.5)	6 (18.18)	1 (10)	
	4	1 (12.5)	13 (39.39)	3 (30)	
Tractional (T)	0	-	-	-	NA
	1	-	-	-	
	2	-	-	-	
	3	-	-	8 (80)	
	4	8 (100)	-	1 (10)	
	5	-	33 (100)	1 (10)	
Neovascular (N)	0	7 (87.5)	29 (87.88)	8 (80)	0.2884
	1	-	-	-	
	2a	-	1 (3.03)	2 (20)	
	2b	1 (12.5)	3 (9.09)	-

**Table 4:** Stratification of ATN classification versus VA gain and anatomical success

ATN classification		VA Gain	*P* value*	Anatomical success	*P* value#
A	Low	20 (76.92)	0.91	19 (73.08)	1
	High	15 (60.00)		20 (80.00)	
N	Low	30 (68.18)	0.39	33 (75.00)	1
	High	5 (71.43)		6 (85.72)	

A: low = 0-2, high =3-4; N: low = 0-1, high = 2a and 2b

T classification was not used for analysis as it represented the individual diagnosis

*Chi-square test, #Fisher exact test

When comparing the first 6-year period (2009-2014) with the second 6-year period (2015-2020), there was a significant increase in cases from 18 to 33. (Kendall Tau b test, correlation coefficient=0.61, 2 tailed p = 0.01). (Table 5)

**Table 5:** Distribution of MTM cases over the 2 periods

	myopic macular hole with no detachment (MH group)	myopic macular hole with retinal detachment (MHRD group)	myopic foveoschisis with retinal detachment (MFRD group)	Total number of cases
Number of cases 2009-2014	2	13	3	18
Number of cases 2015-2020	6	20	7	33

## Discussion

The reported prevalence of MTM ranges widely from 9% to 24% in high myopia and may vary according to the values of axial length and spherical equivalent being adopted as inclusion criteria [4, 14]. It has also been pointed out that only studies that used OCT or colour fundus photography to ascertain the presence of myopic degenerative changes are relevant to gauge the prevalence of myopic foveoschsis.

In the present study, we included 51 cases of MTM over 12 years in a myopia prevalent region. We reported a significant age difference between the MH, MHRD, and MFRD groups (58.6 vs 64.2 vs 66.6). This is in line with other studies that showed that MTM is age-dependent [1, 13]. The younger age of the MH group could be explained by observations that with time, scleral elongation and anteroposterior vitreous traction could cause FTMH to progress to MHRD and the progression of macular-schisis to MFRD in the absence of macular hole. The formation of an FTMH from a normal foveal phenotype was hypothesised to be secondary to the tangential traction due to scleral enlargement, vitreoretinal interface traction and adhesions and happened earlier than MFRD and MHRD [1, 13, 18, 19]. There was no statistical difference in the mean axial length among the three groups (MH 28.4, MHRD 29.6 and MFRD 29.8mm, p=0.08), but the two groups with retinal detachment appeared to have slightly longer axial length. Although we did not detect any difference in the anatomical success rate, it was interesting to see that visual improvement in both MHRD and MFRD groups but not in the MH group. However, this could not be explained by the underlying atrophic changes in which the MHRD and MFRD groups had higher grading of atrophic changes than the MH group. Even after analysis using the ATN classification, we did not observe any correlation with anatomical and visual outcomes.

Most current literature focuses on the anatomical success and outcome of MTM. There appears to be a lack of studies looking into the longitudinal change in the number of surgically operated MTM cases. Our study evaluates the change in demand for surgically operated myopic maculopathies over 12 years. The strength of our study included adopting strict inclusion criteria of axial length equal to or great than 26.50mm. This represented true high myopes as cases of index myopia contributed by the lens were excluded. Whereas in some previous studies, high myopia was defined solely on spherical equivalent greater than -8 dioptres and might have included cases that were not true high myopes. Other strengths included analysis using the recently proposed ATN classification. All of our patients had OCT performed preoperatively to confirm the diagnosis and postoperatively to confirm surgical success.

Like many parts of the world, Hong Kong saw a rise in the post-war population of baby boomers (born between 1946 and 1964) [20]. The proportion of elderly persons aged 65 and above in the total population in Hong Kong rose from 12% in 2006 to 16% in 2016 [20]. In a recent local study in Hong Kong, 72.7% of parents of 4,257 children are myopic [21]. The Beijing Eye study reported that the prevalence of myopic maculopathy in 4,439 adults older than 40 years could be as high as 3.1% [9]. The trend of the growing prevalence of myopia and its complications is worrying.

We observe a significant increase in the number of surgically operated myopic maculopathies over 12 years in a myopia prevalent region. With baby boomers entering retirement, our results may suggest an increasing demand for surgery for myopic maculopathies. Coincidentally, Nielsen et al. recently published the Danish national registry on rhegmatogenous retinal detachment and reported a greater than 50% increase in the prevalence of cases over a ten-year period [22]. Although they could not confirm whether myopia was responsible for the surge of patients, their observation appears consistent with ours in that there is an increase in the demand for vitreoretinal surgeries.

A workforce census by the Royal College of Ophthalmologists in the United Kingdom in 2018 estimated a 30-40% increase in service demand over the next 20 years due to rising population age and new treatment modalities [23]. The current number of graduates from the training programme cannot fulfil the demand. It also appears that the impact of the increasing prevalence of myopia might not have been considered. The Association of American Medical Colleges also predicts a similar trend in the ophthalmology workforce in the US [24]. As the workforce shortage has been focused more on glaucoma, medical retinal and paediatrics, we, as vitreoretinal specialists, should start voicing out to our respective health authorities. Otherwise, additional resources may be channelled to other subspecialties. Workforce planning needs to be started early, as studies on ophthalmology workforce, for example that in relation to cataract surgery in Canada, looked at a time frame of the next 25 years [25].

Nevertheless, we acknowledge certain weaknesses in the present study, including its retrospective nature and relatively small sample size, non-universal use of fundus photographs for grading, and surgeons' discretion on the choice of tamponade agent. Also, there could be an underestimation of the actual prevalence of MTM as we did not include cases that declined surgical intervention. The expansion of surgical volume was also contributed by other factors that could result in selection bias, including increased referrals, easing access to care, and further surgeons’ experience performing delicate manoeuvres. Furthermore, our results may not be generalisable because all cases are of Chinese ethnicity. Further studies with a larger sample size may provide more evidence for our conclusions.

In conclusion, we observe a significant increase in surgically operated myopic maculopathies over 12 years in a myopia prevalent region. With baby boomers entering retirement and a global rise in the prevalence of myopia, it may be a good time to start workforce and service planning for vitreoretinal surgical service. A more extensive study is warranted to look for similar trends in other ethnic groups and countries.

## Data Availability

The datasets used and analysed during the current study available from the corresponding author on reasonable request.
